# Polysomnographic Assessment of Effects of Tobacco Smoking and Alcohol Consumption on Sleep Bruxism Intensity

**DOI:** 10.3390/jcm11247453

**Published:** 2022-12-15

**Authors:** Weronika Frosztega, Mieszko Wieckiewicz, Dorian Nowacki, Monika Michalek-Zrabkowska, Rafal Poreba, Anna Wojakowska, Justyna Kanclerska, Grzegorz Mazur, Helena Martynowicz

**Affiliations:** 1 Department of Internal Medicine, Occupational Diseases, Hypertension and Clinical Oncology, Wroclaw Medical University, 213 Borowska St., 50-556 Wroclaw, Poland; 2 Department of Experimental Dentistry, Wroclaw Medical University, 26 Krakowska St., 50-425 Wroclaw, Poland; 3 Department of Human Nutrition, Wroclaw University of Environmental and Life Sciences, 37 Chelmonskiego St., 51-630 Wroclaw, Poland

**Keywords:** sleep bruxism, polysomnography, alcohol, tobacco, smoking, iron, magnesium

## Abstract

Background: Sleep bruxism (SB) is a common sleep-related movement behavior with a complex etiology. A recent hypothesis suggests psychoactive substance usage, tobacco smoking, and alcohol intake are risk factors for SB. This study aimed to evaluate SB intensity in tobacco smokers and alcohol drinkers. Methods: A total of 133 adults underwent full-night audio- and video-polysomnography, and the polysomnograms were evaluated using the American Academy of Sleep Medicine guidelines. The study group was divided into smoker and nonsmoker groups as well as drinker and non-drinker groups. Results: The results of the polysomnographic analysis confirmed that tobacco smoking has a significant effects on SB. Tobacco smokers showed increased bruxism intensity (5.50 ± 4.71 vs. 3.83 ± 3.26, *p* < 0.05), especially the mixed phenotype (0.93 ± 1.00 vs. 0.59 ± 0.59, *p* < 0.05), in the N1 sleep stage (22.84 ± 20.45 vs. 15.66 ± 13.60, *p* < 0.05) and the nonsupine position (4.93 ± 5.56 vs. 2.50 ± 2.31, *p* < 0.05). They also showed a higher number of bruxism episodes with arousal compared with nonsmokers (2.91 ± 2.83 vs. 1.61 ± 1.49, *p* < 0.05), indicating increased sleep fragmentation. However, no significant effect of alcohol on SB intensity was observed, and the bruxism episode index was similar in alcohol drinkers and nondrinkers. In addition, electrolyte disturbances and lipid disorders were evaluated. Compared with nonsmokers, tobacco smokers showed a higher concentration of plasma triglycerides (177.67 ± 106.9 vs. 129.18 ± 65.61) and lower levels of iron and magnesium (96.68 ± 43.58 vs. 123.83 ± 52.36 and 1.85 ± 0.22 vs. 1.96 ± 0.21, respectively). Conclusions: In summary, this study showed that tobacco smoking, but not alcohol consumption, is related to bruxism intensity and lipid and electrolyte disturbances in individuals with sleep disorders.

## 1. Introduction

Sleep bruxism (SB) is a major sleep-related movement behavior with a complex etiology and an uncertain and insufficiently understood pathophysiology [1].Various causes of SB have been reported, among which genetic contribution is one of the primary subjects of research [2]. Furthermore, the autonomic nervous system has been reported to play a role in SB genesis [3]. The prevalence of SB is estimated between 8% and 31.4% [4]. To date, many attempts have been made to establish a comprehensive definition of bruxism that covers all aspects of this extensive topic; thus, the definition has been evaluated taking into account the growth of knowledge on this subject.

As bruxism has become an emerging problem worldwide and attracted research interest in the medical field, Lobbezoo et al. established a new definition of bruxism, which was extended without the prior restrictions of earlier definitions [5]. They defined SB as “a masticatory muscle activity during sleep that is characterized as rhythmic (phasic) or non-rhythmic (tonic) and is not a movement disorder or a sleep disorder in otherwise healthy individuals”. A possible sleep bruxism diagnosis can be based only on a positive self-report, and a probable bruxism outcome is based on a positive clinical inspection with or without a positive self-report. To consider the outcome as definite, results need to be based on positive instrumental assessment, with or without a positive self-report and/or positive clinical inspection. In general, SB is considered not a medical condition in healthy individuals but more as a harmful or protective behavior, depending on its health outcomes [5]. According to the International Classification of Sleep Disorders, the third edition, bruxism is defined as a “sleep-related movement disorder characterized by teeth grinding or clenching associated with an excessive sleep arousal activity” [6]. No significant association between gender and SB has been observed since bruxism affects females and males equally; thus, gender should be not considered a risk factor for SB [7,8].

Symptoms of SB such as jaw clenching and tooth grinding are risk factors for several health problems [9]. Masticatory muscle activity can lead to masticatory muscle hypertrophy, indentations on the tongue or lip, linea alba on the inner cheek, damage to dental hard tissues (e.g., cracked teeth), repetitive failures of restorative work/prosthodontic constructions, mechanical wear of teeth, masticatory muscle pain, and/or morning headache [9,10]. However, some studies suggest that SB and temporomandibular disorders are less related [11,12,13]. Recently, an association between simple snoring and SB has been reported [14].

SB episodes can be analyzed using polysomnographic examination—the gold-standard tool for SB diagnosis—which is based on night video and audio recordings, along with bioelectric signals from electroencephalograms, electromyography, electrocardiograms, and airflow detectors [15,16].

Cigarettes and alcohol are legal psychoactive substances that affect the central nervous system (CNS) and can also affect the behavior, cognitive functions, and mood of individuals who consume them. Alcohol contains ethanol, which is a CNS depressant that acts as a gamma-aminobutyric acid receptor agonist and an N-methyl-D-aspartate receptor antagonist. Tobacco products contain nicotine, which is an acetylcholine agonist that can stimulate or depress the CNS depending on the amount consumed [4]. The most recent hypotheses of SB etiology imply a putative role of the central and autonomic nervous systems in the development of oromandibular activity during sleep [1]. Tobacco smoking can lead to significant health problems, and tobacco smokers are more likely to experience sleep-related disorders such as poor sleep quality, insomnia, and sleep apnea [17]. A wide-range cohort study of a Finnish adult population reported smoking as an independent risk factor for weekly bruxism, defined as bruxism that occurred frequently within a week [18]. Moreover, smoking is a well-established risk factor for premature mortality and morbidity [19]. In the most recent Global Burden of Disease study published in *The Lancet* in 2019, the major risk factor for death in countries with a middle to high socio-demographic index is the usage of tobacco products, which causes, among other effects, high systolic blood pressure, dietary risks, overweight, and high fasting plasma glucose levels. The primary risk factors have changed from 1990 to 2019, but the pattern remains comparable. In the 25–49 year age group, alcohol use is the most common risk factor, but smoking has become a less significant risk factor and has steadily declined, mainly due to widespread government actions (taxation, regulatory policies for tobacco smoking) that resulted in a behavior change in populations [20]. The association between SB and the usage of legal psychoactive substances has been previously demonstrated [21]. Recently, Michalek-Zrabkowska et al. reported that young individuals with SB tend to have a high cardiovascular risk [22]. Cardiovascular risk is attributable to several modifiable and nonmodifiable risk factors. Tobacco consumption is classified as a modifiable risk factor. Huge efforts are required to change the hazardous habits of patients, including encouraging them to quit smoking [23,24,25].

An unhealthy lifestyle, including the consumption of stimulants, can lead to significant health problems that affect the general health of patients and is a risk factor for bruxism.

Based on this knowledge and due to the lack of data on the cause–effect relationship, the effects of psychoactive substances on SB intensity were hypothesized in this study. The null hypothesis was that increased bruxism intensity occurs in tobacco smokers and alcohol drinkers compared with controls. The aim of the study was to investigate the relationship between bruxism intensity in tobacco smokers and alcohol drinkers using polysomnographic assessments. An additional aim of the study was to evaluate the lipid profile and concentrations of electrolytes and iron in blood samples of tobacco smokers and alcohol drinkers.

## 2. Materials and Methods

This was a prospective, observational study carried out on 133 adult Caucasian individuals in the Sleep Laboratory of the Department and Clinic of Internal Medicine, Occupational Diseases, Hypertension and Clinical Oncology at Wroclaw Medical University. This study was approved by the Ethical Committee of Wroclaw Medical University (no. KB-790/2022). All patients signed voluntary informed consent prior to their participation in the study.

Among the 133 participants, 62% (*n =* 82) used legal stimulants, including tobacco (*n* = 24) and alcohol (*n* = 77). Those who consumed both alcohol and tobacco contributed to 14% of the study group (*n* = 19). Alcohol drinkers who did not smoke contributed to 44% of the study group, and smokers who did not drink contributed to 4% of the study group. The following comorbidities were observed among the participants: myocardial infarction (*n* = 7), stroke history (*n* = 6), hypertension (*n* = 52), diabetes (*n* = 24), and ischemic heart disease (*n* = 10). The characteristics of the study group are presented in Table 1. 

### 2.1. Inclusion and Exclusion Criteria

**Figure 1:**
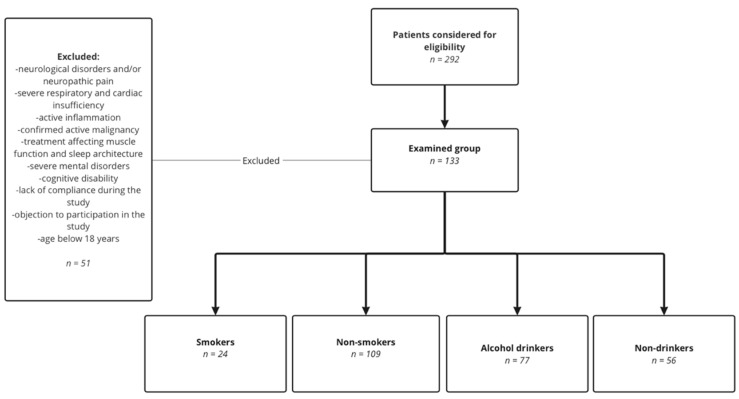
Flowchart showing the study design.

Among 292 patients admitted to the Sleep Laboratory due to suspicion of SB and/or sleep apnea between May 2020 and December 2021, 133 patients were included in the study group based on the following inclusion and exclusion criteria. The inclusion criteria included adults over 18 years of age and the provision of voluntary informed consent to be part of this study. The exclusion criteria were as follows: presence of neurological disorders and/or neuropathic pain, severe respiratory and cardiac insufficiency, active inflammation, confirmed active malignancy, treatments affecting muscle function and sleep architecture, severe mental disorders, cognitive disability, and lack of compliance during the study. The study design is presented in Figure 1.

### 2.2. Study Methods and Design

All patients underwent PSG examination under medical prescription, completed the self-reported questionnaire regarding the usage of legal stimulants such as alcohol drinking and tobacco smoking, and were medically examined. Lifetime smoking was estimated in pack-years, i.e., the number of years a full pack of cigarettes (20 cigarettes) was smoked per day. Smoking status was categorized as smoker or nonsmoker. There were no former smokers in the study group, and all of the smokers were current smokers. Patients were classified as alcohol drinkers or nondrinkers based on self-reporting of any amount of alcohol consumption. Patients were divided into four groups: smokers *n* = 24, nonsmokers *n* = 109, alcohol drinkers *n* = 77, and non-drinkers *n* = 56. The group of patients who declared no legal stimulant usage consisted of 51 patients.

To confirm SB diagnosis and investigate sleep architecture, an instrumental approach was followed using video-polysomnography (PSG) with a NoxA1 (NOX Medical, Reykjavík, Iceland) device. Recordings were made between 22:00 and 06:00, in accordance with the natural circadian rhythm of the patients. Electromyographic electrodes were placed according to the recommendations of the American Academy of Sleep Medicine (AASM). The PSG examination included video and audio recording, along with electrocardiographic, electroencephalographic, electrooculographic, and electromyographic recordings (origin signal from the chin and masseter muscles), body position detection, and thoracic and abdominal breathing activity. Saturation level (SpO2%), pulse, and plethysmography data were recorded using a NONIN WristOx2 3150 pulse oximeter (Nonin Medical Inc., Plymouth, MN, USA). The recordings were stored using Noxturnal software (Nox Medical, Reykjavík, Iceland). The obtained PSG recordings were analyzed and interpreted in 30 s epochs in accordance with the guidelines for sleep scoring by a qualified physician in the Sleep Laboratory [26].

Rhythmic activities of masseter muscles occurring at least 3 s after the previous muscle activity were recognized as SB episodes. These episodes were frequently accompanied by specific movements in the orofacial region and grinding sounds. The bruxism episode index (BEI) was used to measure the bruxism intensity by counting the number of bruxism episodes per hour of sleep. Three phenotypes of SB were distinguished using electromyographic assessments: (1) phasic, characterized by ≥3 bursts of muscle contractions at 1 Hz frequency; (2) tonic, characterized by a contraction that lasts >2 s; and (3) mixed, including both phasic and tonic movements [27]. After overnight polysomnographic examinations, blood samples were obtained from the patients by venipuncture at 7 a.m., after 12 h of overnight fasting, which were analyzed at the Main Laboratory of Wroclaw Medical University. Lipid profile analysis and other laboratory tests were conducted in accordance with the standard laboratory protocols of Wroclaw University Teaching Hospital.

The sample size was evaluated using a sample size calculator based on the following standard assumptions: population size, 3,000,000; population proportion, 8%; confidence level, 0.95; and maximum error, 5%. The required group size was found to be 114. Thus, a larger group (*n* = 133) was recruited in the present study.

### 2.3. Data Analysis

Statistical analysis of the data was carried out using the statistical analysis program Statistica 13.3 (Statsoft, Krakow, Poland). The data are presented as the mean ± standard deviation. Differences between the groups were analyzed using Student’s *t*-test or analysis of variance (ANOVA) in the case of parametric data and the Mann–Whitney U test or Kruskal–Wallis ANOVA in the case of nonparametric data. Correlations were evaluated using Pearson correlation coefficients. A *p* value < 0.05 was considered to be statistically significant.

## 3. Results

The average age of the study population was 47.89 ± 16.57 years, with women comprising 45% (*n* = 60) of the study group and men comprising 55% (*n* = 73). The mean pack-years in the smoker group was 13.60 ± 2.77. The polysomnographic parameters are presented in Table 2 and Table 3.

Major differences were observed between the smoker and nonsmoker groups. The PSG examination showed that smokers had a statistically significantly higher BEI than nonsmokers. Bruxism episodes were more frequent in smokers during the night in non-rapid eye movement sleep, especially during the N1 sleep stage and in the nonsupine position, compared with nonsmokers. The frequency of mixed episodes was also increased in smokers. The number of bruxism episodes with arousal was significantly higher in smokers than in nonsmokers (Table 2).

Statistically significant positive correlations were observed between pack-years and BEI (*r* = 0.61, *p* = 0.002), phasic BEI (*r* = 0.62, *p* = 0.002), mixed BEI (*r* = 0.44, *p* = 0.033), tonic BEI (*r* = 0.42, *p* < 0.041), and bruxism with arousal (*r* = 0.50, *p* = 0.012).

A statistically significantly higher plasma triglyceride (TG) content was observed among smokers compared with nonsmokers; however, no significant differences were observed between alcohol drinkers and nondrinkers. Noticeable changes were observed in the electrolyte and blood composition of smokers, which were characterized by lower magnesium (Mg) and iron (Fe) levels in the blood. No statistical differences were observed in sodium, potassium, calcium, total cholesterol, low-density lipoprotein, and high-density lipoprotein levels in smokers compared with nonsmokers. The blood parameters are presented in Table 4.

## 4. Discussion

Although many research studies on SB are available in the literature, the majority of studies are based only on non-instrumental approaches using self-reported or telephone surveys [18,26,28,29,30,31,32,33].

In an international consensus, Lobbezoo et al. established that an instrumental approach for PSG assessment is the gold-standard for “definite” diagnosis of SB [5]. Non-instrumental approaches such as questionaries can only determine a “possible” diagnosis and further confirmation using clinical investigation and PSG examination is required [34]. The present study was based on PSG examinations, thus fulfilling the “definite” SB diagnosis requirements.

### 4.1. Effects of Smoking on SB

The most important finding of this study was the higher BEI in smokers compared with nonsmokers. Taking into account the electromyographic phenotype of episodes, a higher number of mixed bruxism episodes was observed in smokers compared with nonsmokers. As reported in a previous study, bruxism episodes are strongly associated with the sleep stages of non-rapid eye movement sleep, including the N1 and N2 stages, which are light sleep stages [35]. To the best of our knowledge, this study is the first research showing that tobacco smoking increases BEI during the N1 stage of sleep and in the nonsupine sleep position.

According to the AASM guidelines, arousals are “sleep perturbations characterized by a transient increase (for 3–15 s) in electroencephalography fast wave activity, with or without an increase in EMG activity and cardiac rhythm” [35,36]. Sleep arousal can be classified as respiratory arousal, which is associated with respiratory events, and movement arousal, which is accompanied by body movements (SB, PLM). Several SB mechanisms have been reported to date, and, among many etiological concepts, arousal is considered a trigger for RMMA [26,37]. Arousal in the context of SB is highly associated with the term “sleep fragmentation,” along with wake after sleep onset (WASO) [38]. The impact of SB on sleep quality remains a subject of research, and many studies suggest that SB does not directly influence sleep quality or that it slightly lowers sleep quality [39,40]. In the present study, smokers showed an increased intensity of bruxism with arousal, suggesting increased sleep fragmentation, which is a novel and interesting finding. Very few research studies have used diagnoses based on or supported by instrumental approaches [41,42,43]. Moreover, no study has investigated the electromyographic phenotype and influence of arousal and body position during sleep in smokers and drinkers. Lavigne et al. established that smokers grind their teeth more frequently than nonsmokers in a polysomnographic study carried out on a Canadian population [44]. In the present study, smokers showed a higher mixed muscle activity (mixed episodes) than nonsmokers. These findings are in accordance with those of previous studies, pointing out that more than 88% of SB episodes confirmed using vPSG were of phasic or mixed type [45,46].

To the best of our knowledge, this is the first study to use PSG in a large study group (*n* = 133) to analyze SB-related movements and sleep architecture in smokers and alcohol drinkers. These results support the findings of previous studies, indicating that SB can be related to smoking [7,18,26,44,47,48,49,50,51,52,53]. However, some studies have failed to establish the association between these factors. Wincour et al. reported that no correlation between smoking, drinking, and SB in Israeli adolescents. Their study was limited by a small smoker group and the fact that alcohol consumption is the lowest in Israel among OECD countries [33]. Another study reported a negative association between tobacco smoking and SB but a positive association with awake bruxism among Dutch adolescents [30].

In hypertensive patients, the risk of SB tends to be increased with concomitant smoking and they experience a high number of bruxism episodes [33]. Martynowicz et al. stated that along with BEI, other risk factors for increased BEI include a high body mass index, a lower mean oxygen saturation (SpO2), and a higher percentage of SpO2 < 90% [54]. A recent study indicated that levels of inflammation markers were significantly increased by SB [22]. Furthermore, inflammation in the endothelium and atherosclerosis development due to the deleterious influence of SB was reported to be a risk factor for hypertension [22]. Many researchers have reported the influence of smoking on inflammation. The proinflammatory influence of smoking on increased levels of inflammation markers and cytokines has also been reported [55,56,57]. Thus, hypertension and smoking are risk factors for SB and lead to increased inflammation, which is associated with the risk of cardiovascular diseases.

A previous study on a large group of patients with SB confirmed that SB impairs sleep architecture, affects the total sleep time, NREM N3 time, NREM sleep latency, sleep efficiency (SE), and increases the index of microarousals [58]. Furthermore, another study provided further knowledge about differences in sleep structure [59]. In the present study, a significantly higher number of bruxism episodes were observed in smokers in the nonsupine position (BEI nonsupine). The influence of sleep position on SB has rarely been discussed in previous studies, although one study investigated the influence of sleep position on SB occurrence [60]. Recently, Michalek-Zrabkowska et al. reported the dependence of sleep position on SB. The nonsupine position has been found to be correlated with a higher number of bruxism episodes and desaturation [14]. To the best of our knowledge, no other study has investigated the relationship between sleep position and bruxism episodes in smokers.

Several studies have reported the deleterious effects of tobacco on general health. A previous study investigated the influence of tobacco smoking on iron homeostasis dysregulation [61]. Another recent study reported a lower iron content due to smoking [62]. Smoking is a known and underestimated risk factor for iron deficiency anemia, which is more frequent among light smokers and depends on the smoking exposure time (duration of smoking) [63]. Exposure to tobacco smoke is one of the major risk factors of chronic obstructive pulmonary disease (COPD) [64]. Approximately 50% of heavy smokers tend to develop COPD [65]. This study indicated that the prevalence of nonanemic iron deficiency was higher in COPD patients due to increased levels of inflammation markers. COPD patients tend to have lower blood oxygen levels with no coexisting airflow obstruction [66]. Therefore, in the present study, the lower blood iron content observed in smokers compared with nonsmokers is in accordance with the findings of previous studies.

The effect of smoking on dyslipidemia is a well-researched topic. Increased serum lipid profiles, especially TG, are strongly correlated with obesity and smoking, which is consistent with the findings of the present study [65,67,68,69].

The finding that the magnesium content was lower in smokers is consistent with the findings of previous studies [70,71]. This can be explained by the fact that smokers consume lower amounts of fruits, vegetables, cereals, and dairy. As a result, their magnesium intake could be lower than in nonsmokers [72]. An inverse relationship between inflammation markers and magnesium content in smokers has also been reported in previous studies [70]. Thus, these results are in agreement with the findings of the present study.

### 4.2. Effect of Alcohol Intake on SB

In the present study, no significant differences in bruxism intensity and sleep architecture were observed between alcohol drinkers and nondrinkers. It is well established that alcohol consumption has adverse health effects on health and sleep [73,74]. Alcohol drinkers are at risk of cardiovascular diseases, type 2 diabetes, dementia, cancers, alcoholic liver disease, malnutrition, and several other associated diseases [74]. Alcohol makes falling asleep easier but, on the other hand, disrupts sleep architecture, triggers insomnia, and leads to natural circadian rhythm changes. In individuals with preexisting breathing-related disorders, alcohol consumption worsens respiratory events and lowers the oxygen saturation level [74,75]. Alcohol affects nocturnal sleep parameters such as reduced sleep onset latency, WASO, slow wave sleep in N3, and REM sleep [76]. Only a few studies have investigated the effect of alcohol intake in SB patients using PSG examinations [33]. Holanda et al. reported that alcohol consumption was strongly correlated with SB. The findings of Itani et al. supported the correlation between alcohol intake and smoking habits with SB in a research survey [26]. However, slight differences in WASO and SE were observed between drinkers and nondrinkers, which could be attributable to more consolidated sleep in the first hours of the night [68]. Therefore, alcohol, through sleep consolidation in the first hours of the night, may eliminate arousals and reduce SB. In contrast, in the latter part of the night, alcohol could show the opposite effect and promote SB. However, this hypothesis needs further research.

### 4.3. Study Strengths

This study included a large study sample of 133 patients. Furthermore, this study was based on polysomnography, which is the “gold-standard tool” for SB diagnosis according to Lobbezoo et al. [5]. Moreover, this study measured blood parameters, which added to the characterization of the study population. To the best of our knowledge, this is the first study to use PSG evaluations to determine SB intensity in smokers and drinkers.

### 4.4. Limitations

There were a few limitations to this study. The small size of the smoker group is correlated with a higher risk of errors than in a larger sample group. In addition, patients were admitted to the Sleep Laboratory with no adaptive night before the avPSG examination due to the organization of the Polish health service and the limited capabilities of the laboratory. The group of nonsmokers could include alcohol drinkers, which may cause bias.

## 5. Conclusions

1. Tobacco smokers have a higher bruxism intensity, especially the mixed phenotype, in N1 sleep and in the nonsupine position.
2. Tobacco smokers have a higher number of bruxism episodes with arousal than nonsmokers, which suggests increased sleep fragmentation.
3. Smokers with comorbid sleep-related disorders tend to have electrolyte disturbances and lipid disorders.
4. Alcohol consumption has no significant influence on bruxism parameters.

## Figures and Tables

**Table 1:** The characteristics of the study group.

Parameter	Total (*n* = 133, %)	Smokers (*n* = 24, %)	Nonsmokers (*n* = 109, %)	Alcohol Drinkers (*n* = 77, %)	Nondrinkers (*n* = 56, %)
Myocardial infarction	7, 5%	5, 20.8%	2, 1.83%	5, 6.5%	2, 3.5%
Stroke	6, 4.5%	4, 16.6%	2, 1.83%	4, 5.2%	2, 3.5%
Hypertension	52, 39%	13, 54.2%	39, 35.7%	32, 41.5%	20, 35.7%
Diabetes	24, 18%	7, 29.2%	17, 15.6%	16, 20.7%	8, 14.3%
Ischemic heart disease	10, 7.5%	4, 16.6%	6, 5.5%	6, 7.8%	4, 7.1%

**Table 2:** Sleep bruxism parameters differentiated based on the usage of legal drugs (alcohol and tobacco).

Parameter	Smokers	Nonsmokers	*p*	Alcohol Drinkers	Nondrinkers	*p*
Bruxism episode index (BEI) (n/h)	**5.50 ± 4.71**	**3.83 ± 3.26**	**0.045**	4.07 ± 3.38	4.26 ± 3.96	0.750
Phasic episodes (n/h)	2.88 ± 3.02	1.89 ± 2.27	0.051	2.13 ± 2.23	2.01 ± 2.69	0.370
Tonic episodes (n/h)	1.70 ± 1.31	1.34 ± 1.27	0.130	1.32 ± 1.09	1.53 ± 1.51	0.779
Mixed episodes (n/h)	**0.93 ± 1.00**	**0.59 ± 0.59**	**0.039**	0.62 ± 0.69	0.71 ± 0.72	0.538
BEI supine (n/h)	8.02 ± 8.62	6.74 ± 11.25	0.291	5.70 ± 5.83	8.75 ± 15.05	0.999
BEI nonsupine (n/h)	**4.93 ± 5.56**	**2.50 ± 2.31**	**0.002**	3.20 ± 3.71	2.58 ± 2.50	0.488
BEI N1 (n/h)	**22.85 ± 20.46**	**15.67 ± 13.61**	**0.042**	17.72 ± 15.38	16.10 ± 15.31	0.414
BEI N2 (n/h)	5.34 ± 5.45	3.91 ± 4.20	0.250	3.97 ± 3.87	4.46 ± 5.22	0.912
BEI N3 (n/h)	2.05 ± 2.29	1.58 ± 1.94	0.0540	1.73 ± 2.23	1.59 ± 1.70	0.660
BEI REM (n/h)	3.54 ± 2.50	3.12 ± 2.67	0.340	3.00 ± 2.43	3.48 ± 2.89	0.470
Bruxism with arousal (n/h)	**2.91 ± 2.84**	**1.61 ± 1.49**	**0.000006**	1.84 ± 1.96	1.86 ± 1.73	0.833

BEI: bruxism episode index, REM: rapid eye movement, Statistically significant values are shown in bold (*p* < 0.05).

**Table 3:** Polysomnographic evaluation of the study group.

Parameter	Smokers	Nonsmokers	*p*	Alcohol Drinkers	Nondrinkers	*p*
AHI (n/h)	21.23 ± 26.79	19.52 ± 22.74	0.811	17.28 ± 21.14	23.28 ± 26.00	0.270
ODI (n/h)	21.11 ± 26.23	17.56 ± 20.10	0.861	16.68 ± 19.90	20.28 ± 23.00	0.580
Snore (%)	26.84 ± 24.30	19.49 ± 20.23	0.246	23.01 ± 23.00	17.85 ± 18.10	0.501
PLMS index (n/h)	8.33 ± 13.30	7.96 ± 18.05	0.552	10.10 ± 21.40	5.21 ± 8.50	0.084
SL (min)	18.81 ± 29.75	17.99 ± 18.90	0.460	15.32 ± 13.52	21.96 ± 28.10	0.210
REM latency (min)	97.80 ± 68.10	101.70 ± 80.00	0.899	89.51 ± 56.60	116.42 ± 98.00	0.218
WASO (min)	60.03 ± 47.11	67.12 ± 64.25	0.804	**56.71 ± 60.23**	**78.20 ± 61.32**	**0.007**
SE (%)	79.73 ± 16.81	81.85 ± 13.74	0.581	**83.13 ± 15.00**	**79.21 ± 13.16**	**0.013**
N1 (% of TST)	7.23 ± 5.70	6.71 ± 6.50	0.647	6.53 ± 5.80	7.17 ± 7.00	0.795
N2 (% of TST)	46.19 ± 12.72	50.43 ± 23.60	0.264	47.27 ± 10.51	52.90 ± 31.43	0.498
N3 (% of TST)	24.00 ± 10.10	26.67 ± 27.03	0.701	23.32 ± 8.20	30.10 ± 36.71	0.181
REM (% of TST)	22.60 ± 8.93	22.58 ± 9.93	0.650	22.90 ± 7.50	22.18 ± 12.21	0.345
AI (n/h)	9.55 ± 7.76	6.75 ± 6.34	0.063	7.23 ± 7.05	7.30 ± 6.19	0.732

Statistically significant values are shown in bold (*p* < 0.05).

**Table 4:** Comparison of blood parameters regarding tobacco smoking and alcohol drinking.

Parameter	Smokers	Nonsmokers	*p*	Alcohol Drinkers	Nondrinkers	*p*
Mg [mmol/L]	**1.85 ± 0.22**	**1.96 ± 0.21**	**0.042**	1.95 ± 0.21	1.93 ± 0.23	0.417
Fe [µg/dL]	**96.68 ± 43.58**	**123.83 ± 52.36**	**0.041**	121.55 ± 48.87	113.46 ± 55.15	0.408
Na [mmol/L]	139.92 ± 1.72	139.90 ± 2.35	0.688	139.90 ± 2.35	139.92 ± 1.71	0.703
K [mmol/L]	4.31 ± 0.26	4.29 ± 0.31	0.685	4.29 ± 0.28	4.30 ± 0.33	0.913
Ca [mmol/L	9.32 ± 0.31	9.33 ± 0.31	0.752	9.31 ± 0.28	3.36 ± 0.34	0.216
Total cholesterol [mg/dL]	212.73 ± 56.63	196.80 ± 46.31	0.154	204.52 ± 49.00	193.16 ± 47.55	0.148
LDL [mg/dL]	124.86 ± 47.20	112.60 ± 39.16	0.317	119.38 ± 40.66	108.33 ± 40.48	0.154
HDL [mg/dL]	54.59 ± 16.42	57.43 ± 15.64	0.459	56.84 ± 15.94	56.98 ± 15.64	0.804
TG [mg/dL]	**177.68 ± 106.96**	**129.18 ± 65.61**	**0.016**	143.69 ± 76.02	130.81 ± 78.39	0.066

TG: triglycerides, Mg: magnesium, Fe: iron, Na: sodium, K: potassium, Ca: calcium, LDL: low-density lipoprotein, HDL: high-density lipoprotein, statistically significant values are shown in bold (*p* < 0.05).

## Data Availability

The data supporting the findings of this study are available on request from the corresponding author and are not publicly available due to privacy or ethical restrictions.
